# Cytoreductive chemotherapy in induction therapy plays a key role in the prognosis of patients with low‐risk acute promyelocytic leukaemia

**DOI:** 10.1111/jcmm.18252

**Published:** 2024-05-20

**Authors:** Xiaolu Zhu, Feifei Tang, Wenjing Yu, Xiaosu Zhao, Yazhen Qin, Qian Jiang, Xiaojun Huang, Hao Jiang

**Affiliations:** ^1^ Peking University People's Hospital, Peking University Institute of Hematology, National Clinical Research Center for Hematologic Disease, Beijing Key Laboratory of Hematopoietic Stem Cell Transplantation, Peking University Beijing China; ^2^ Peking‐Tsinghua Center for Life Sciences, Academy for Advanced Interdisciplinary Studies Peking University Beijing China

**Keywords:** acute promyelocytic leukaemia, cytoreductive chemotherapy, etoposide, low risk, relapse

## Abstract

In order to explore the risk factors of relapse and potential optimized therapeutic regimen of low‐risk acute promyelocytic leukaemia (APL), here we retrospectively analysed 282 patients who were diagnosed between February 2014 and September 2021. The median follow‐up was 59 (9–102) months. The 5‐year overall survival and cumulative relapse incidence were 97.9% and 5.9%, respectively. In terms of different cytoreductive therapies, 86 patients were administered with hydroxycarbamide (30.5%), 113 with anthracyclines or cytarabine (40.1%), 31 with etoposide (11.0%) and 52 with no cytoreductive therapy (18.4%) during the induction therapy. The hydroxycarbamide treatment group did not decrease the relapse rate compared to the no cytoreduction group (11.4% vs. 5.9%, *p* = 0.289). Compared with the hydroxycarbamide group, the anthracyclines/cytarabine treatment group showed improved 5‐year RFS (88.145% vs. 98.113%, *p* = 0.008). Multivariate Cox regression analysis revealed that myeloblasts in bone marrow at diagnosis, and *PML‐RARA* transcript level of 6.5% or more after induction therapy were associated with a subsequent risk of relapse. The only factor positively reducing the relapse rate was anthracyclines/cytarabine cytoreductive treatment. In conclusion, cytoreductive chemotherapy in induction therapy plays a potential key role in the prognosis of low‐risk APL.

## 1 INTRODUCTION

Despite the success of low‐risk acute promyelocytic leukaemia (APL) in the all‐trans retinoic acid (ATRA) plus arsenicals era, several important clinical issues continue to account for treatment failure including early death (ED) and disease relapse. The ED rate in low‐risk APL was less than 4%.^1^, ^2^, ^3^, ^4^, ^5^, ^6^ Differentiation syndrome (DS), which is a common side‐effect of differentiating agents during induction treatment, occurred in 11%–28% of patients with low‐risk APL.^2^, ^7^, ^8^ Several studies demonstrated that leukocytosis increases susceptibility of DS and even ED.^9^ Approximately 70% of low‐risk patients treated with arsenicals develop leukocytosis with induction therapy, with a median peak white blood cell (WBC) count of 20 × 10^9^/L at about 10 days from the start of treatment.^7^ Thus, the efficient and safe reduction of WBC count by cytoreductive therapy is the key point in the induction therapy of low‐risk APL. Guidelines from the National Comprehensive Cancer Network (NCCN) recommended hydroxyurea, anthracycline or gemtuzumab ozogamicin (GO) for cytoreduction.^10^ Limited effectiveness of hydroxyurea, inconvenient intravenous injection of anthracycline or unavailability of GO in China hampered the progress of the therapeutic regimens for APL.

The combination of ATRA and ATO resulted in statistically significant better EFS and OS rates, reduced cumulative incidence of relapse and lower toxicity, compared to ATRA plus chemotherapy.^2^, ^3^ While previous studies by our group and others showed a relapse of 1.0%–4.8% for low‐risk APL, and the median time to haematological relapse was 20.5 months after a haematological complete remission (CR).^2^, ^5^ We and others have indicated that the addition of cytarabine in induction therapy might correlate with a lower relapse rate.^5^, ^11^, ^12^ Whether cytoreduction in induction therapy in the setting of ATRA plus arsenicals has prognostic significance in APL, besides its role in leukocytosis, remains unclear.

Etoposide is an antitumour inhibitor of topoisomerase II widely used in the treatment of several malignant haematological tumours. The successful experience in two high‐risk APL patients demonstrated the efficacy, safety and convenience of oral etoposide as an alternative cytoreductive agent at the initial stage of induction therapy.^13^ Therefore, the present study was conducted to explore the potential role of cytoreduction during induction therapy on prognosis, and further exploit the all‐oral induction regimen for low‐risk APL with etoposide combined with ATRA plus realgar‐indigo naturalis formula (RIF), an oral realgar (As₄S₄)‐containing formula, as the front‐line therapy for low‐risk APL.

## 2 MATERIALS AND METHODS

### 2.1 Patients

All consecutive low‐risk APL patients who received ATRA plus arsenic for induction and consolidation therapy at the Peking University People's Hospital, Peking University Institute of Haematology, between February 2014 and September 2021, were analysed. Low‐risk patients were defined as a WBC count of no more than 10 × 10^9^/L at diagnosis.^10^ Between February 2014 and August 2015, low‐risk APL patients randomly received RIF‐ATRA or arsenic trioxide (ATO)‐ATRA for induction and consolidation therapy as part of a randomized, controlled phase III clinical trial (ChiCTR‐TRC‐13004054) and data of 59 patients was reported in our previous study.^4^ Between September 2015 and March 2018, low‐risk APL patients were treated with intravenous or oral arsenic based on patients' choice and data from 153 patients was reported in our previous study.^5^ After April 2018, low‐risk APL patients were treated with RIF plus ATRA as a front‐line therapy recommended by the ‘Chinese Guidelines for Diagnosis and Treatment of Acute Promyelocytic Leukemia (2018)’.^14^ Approval was obtained from the Ethics Committee of Peking University People's Hospital (No.2022PHB342‐001), and written informed consent was obtained from all patients according to the Declaration of Helsinki.

### 2.2 Treatment procedure^4^, ^14^

All‐trans retinoic acid (25 mg/m^2^/day divided into two oral doses) was administered immediately upon suspicion of APL based on morphological features. RIF (60 mg/kg/day divided into three oral doses) or ATO (one intravenous dose of 0.15 mg/kg/day) was administered upon detection of the *PML‐RARA* fusion gene via reverse‐transcription polymerase chain reaction (RT‐PCR) or the t(15;17) translocation via conventional karyotyping or fluorescence in situ hybridization. Since genetic confirmation of the diagnosis takes 5 to 7 days, the arsenic therapy was administered approximately 5 to 7 days later than ATRA. RIF or ATO plus ATRA was maintained until CR was reached after induction therapy.

Cytoreduction therapy, including hydroxycarbamide, anthracyclines and cytarabine during induction therapy was administrated based on the 2018 Chinese APL guidelines.^14^ Given the unavailability and inconveniences of intravenous chemotherapy, our centre explored the routine of oral etoposide as cytoreductive therapy from January 2020.

Consolidation therapy was conducted in accordance with the 2018 Chinese APL guidelines. We considered two cycles of ATRA and one cycle of RIF or ATO to be one complete round of consolidation therapy, and thus, the patients received a total of approximately four rounds of consolidation therapy.

### 2.3 Definitions of outcomes

Leukocytosis was defined as a white blood cell (WBC) count greater than 10 × 10^9^/L during induction therapy. Haematological CR was defined as a proportion of BM blasts less than 5%, no blasts in Auer rods, no extramedullary disease, an absolute neutrophil count greater than 1 × 10^9^/L and a platelet count greater than 100 × 10^9^/L, with no red‐cell transfusions. Haematological relapse was defined as the recurrence of blasts greater than 5% in the BM, the reappearance of blasts in the blood or the development of extramedullary disease infiltrates in any site. Molecular relapse was defined as a confirmed reversal of *PML‐RARA* positivity within 4 weeks. Complete molecular remission (CMR) was defined as the absence of detectable *PML‐RARA* transcripts by quantitative RT‐PCR. The sensitivity level of RT‐PCR for *PML‐RARA* transcriptions, as evaluated by series dilution experiments, was 10^−4^. Overall survival (OS) was defined as the time from diagnosis to the date of death, regardless of any cause or last follow‐up. Relapse‐free survival (RFS) was defined as the time from CR to relapse (including hematologic and molecular relapses) or death. In the PETHEMA studies, DS severity was graded according to the number of clinical criteria met to predict outcomes. Patients with four or more of the above signs and symptoms were classified as having severe DS, whereas those with less than four signs and symptoms were classified as having moderate DS.^15^, ^16^ Acute and subacute toxicity of anticancer drugs was graded according to the WHO classification standard. During induction therapy and consolidation therapy, common haematological and non‐haematological adverse events were monitored twice per week.

### 2.4 Response evaluation and molecular monitoring

Every bone marrow sample in our institute was tested and reviewed by two independent hematopathologists. Bone marrow (BM) was evaluated for induction therapy at 4–6 weeks after the blood cell count recovered, namely, when the neutrophil absolute count was ≥1.0 × 10^9^/L and the platelet count was ≥100 × 10^9^/L. BM assessments were conducted every 2 months during consolidation therapy. Following consolidation therapy, BM assessment, including morphology, immunophenotyping and *PML‐RARA* transcript detection, was performed every 3 months during the first 2 years and every 6 to 12 months thereafter. Quantitative RT‐PCR was performed to monitor the levels of *PML‐RARA* transcript in BM as previously described.^5^ Definitions of haematological CR, haematological relapse, complete molecular remission (CMR), molecular relapse, overall survival (OS), relapse‐free survival (RFS) and toxic effects are explained as previously described.^5^

### 2.5 Statistical analysis

The Mann–Whitney *U*‐test was performed for continuous variables, and the chi‐squared test was used for categorical data. Kaplan–Meier was used to estimate relapse rates and OS. Log‐rank tests were performed using the Statistical Package for the Social Sciences (SPSS), version 22.0 (SPSS, Inc., Chicago, IL, USA). A *p* < 0.05 was considered statistically significant. Exploration of factors associated with relapse was based on the Cox proportional hazards regression model with R software (version 4.2.0). Risk factors with a *p* < 0.2 in the univariate analysis were selected for further evaluation by multivariate Cox proportional hazard models. Factor leukocytosis was not selected because of its collinearity with the factor of anthracyclines/cytarabine.

## 3 RESULTS

### 3.1 Patients

**FIGURE 1:**
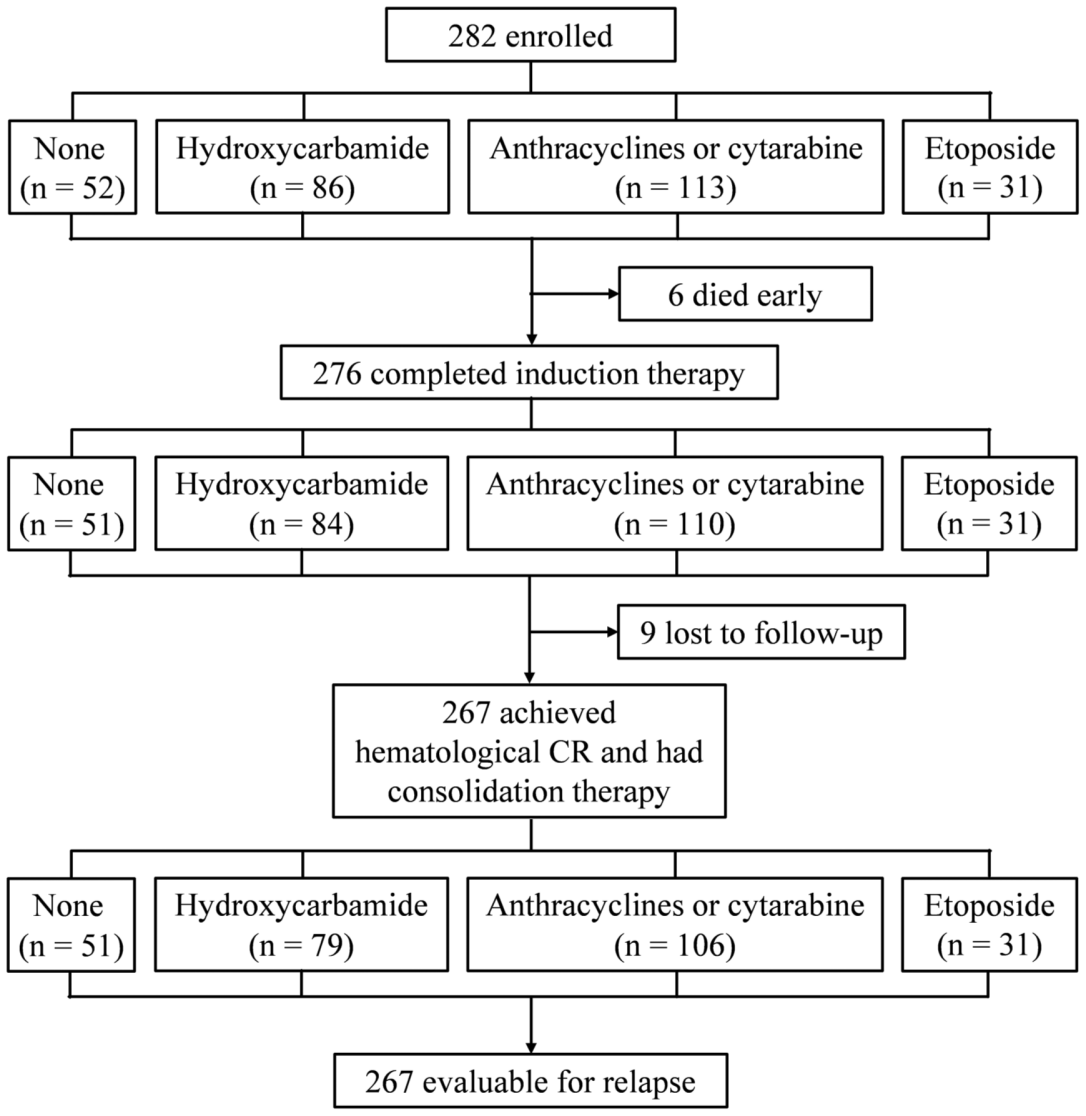
Study diagram. CR, complete remission.

An overview of the study algorithm can be found in Figure 1. From February 2014 to September 2021, 282 patients were diagnosed with low‐risk APL and received ATRA plus arsenic as an induction and consolidation therapy. A total of 246 patients (87.2%) received RIF and ATRA as front‐line induction therapy, and 36 (12.8%) received ATO and ATRA. During induction therapy, six patients died early. Nine patients were withdrawn after induction therapy. Finally, a total of 267 patients were evaluated for relapse. Follow‐up was last conducted on 31 August 2022. The median follow‐up was 59 months [interquartile range (IQR), 41–81; range 9–102]. A summary of the baseline characteristics of the patients can be found in Table S1. The median (range) age of the patients was 39 (13–79) years and 9.2% (26/282) were aged >60 years. There was a higher proportion of males (*n* = 151, 53.5%) and a major *PML‐RARA* subtype of long (*n* = 181, 64.2%). Forty‐five patients (16.0%) were confirmed with complex chromosomal abnormalities and 39 (13.8%) with FMS‐like tyrosine kinase 3‐internal tandem duplication (*FLT3‐ITD*) gene positive at diagnosis.

### 3.2 Treatment response

After induction therapy, all 276 evaluable patients (100%) achieved haematological CR except for six patients who died early. The median (range) time to haematological CR was 36 (28–49) days. Leukocytosis occurred in 165 (58.5%) of the 282 patients during induction therapy. Moreover, the WBC count increased above 4 × 10^9^/L during induction therapy in almost all the patients (263/282, 93.3%). Overall, 49 patients (49/282, 17.4%) developed DS, including moderate and severe forms. Six patients developed ED: three patients died of severe DS, two of cerebral haemorrhage on days 7 and 14, respectively, and one of intracranial infection on day 29 of induction therapy. No patient died during the consolidation therapy.

**FIGURE 2:**
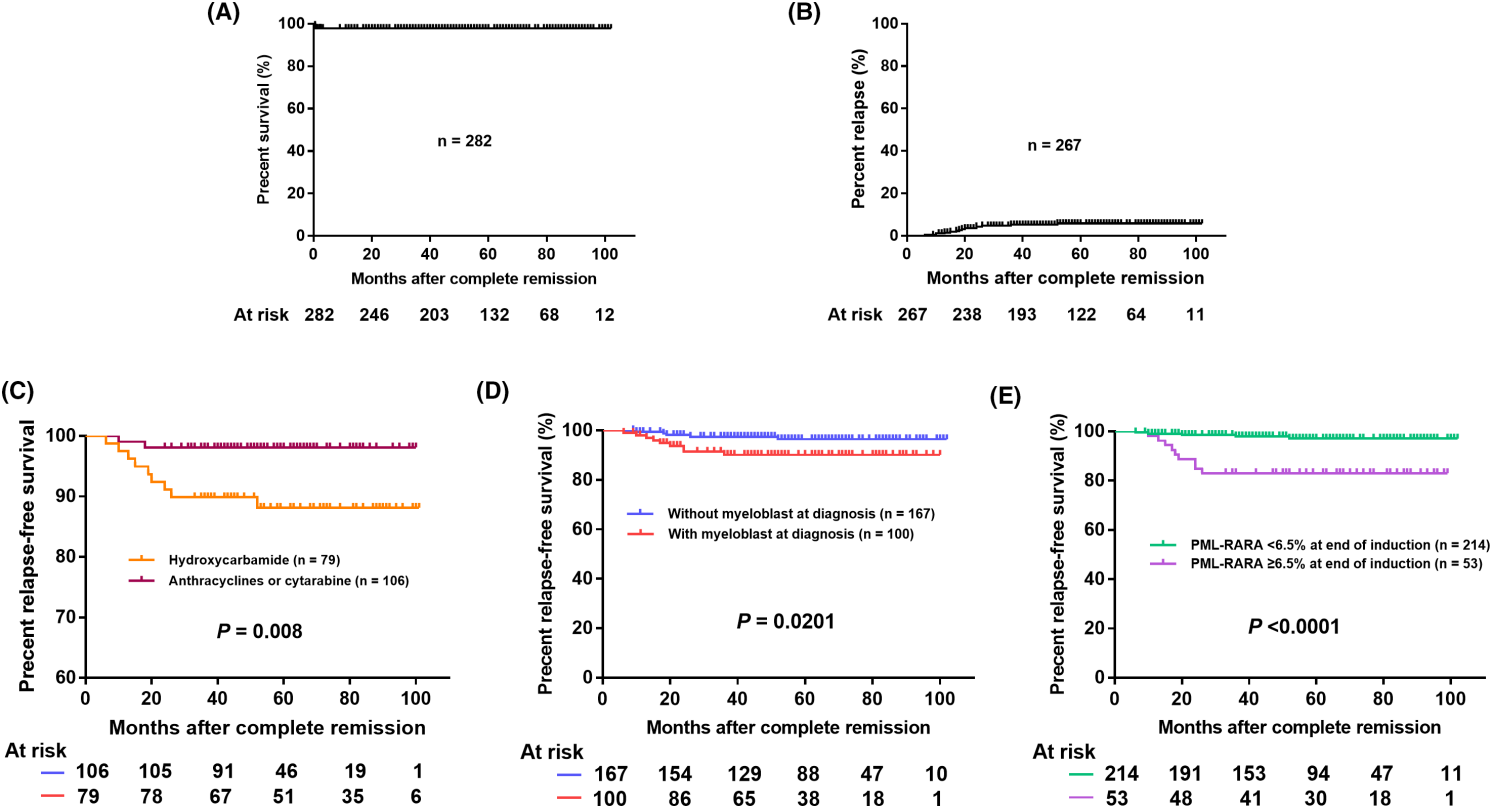
Kaplan–Meier plot of (A) 5‐year overall survival (OS); (B) 5‐year cumulative relapse; (C) relapse‐free survival (RFS) among the hydroxycarbamide and the anthracyclines/cytarabine groups during induction therapy; (D) RFS between with and without myeloblasts in bone marrow at diagnosis; (E) RFS between *PML‐RARA* transcript levels of ≥6.5% and <6.5% after induction therapy.

Of the 282 patients evaluable for OS, the 5‐year OS was 97.9% (Figure 2A). Of the 267 patients evaluable for relapse, 14 patients (5.2%) relapsed throughout follow‐up, with a 5‐year cumulative relapse incidence of 5.9% (Figure 2B). Among them, 12 patients experienced haematological relapse after CMR, one of whom developed central nervous system leukaemia (CNSL). The remaining two patients experienced molecular relapse, one of whom developed CNSL. The median time to relapse, including haematological, molecular and extramedullary relapse, was 18.5 (IQR 12.25–24.5, range 6.2–52.0) months after haematological CR. No death occurred in patients with relapse during the follow‐up.

### 3.3 Cytoreduction therapy during the induction therapy

In terms of different cytoreduction therapies, 86 patients were administered with hydroxycarbamide (30.5%), 113 with anthracyclines or cytarabine (40.1%), 31 with etoposide (11.0%) and 52 with no cytoreductive therapy (18.4%) during induction therapy. Two patients in the no cytoreduction group developed leukocytosis during induction with the maximum WBC count of 13.22 × 10^9^/L and back to less than 10 × 10^9^/L within 1 day without any cytoreduction therapy. Hydroxycarbamide was initiated in patients with a WBC count of 4.31–10.45 × 10^9^/L during induction. Leukocytosis during induction occurred in 43 (50.0%) patients with the maximum WBC count of 17.36 × 10^9^/L. Anthracyclines or cytarabine was initiated in patients with a WBC count of 9.18–13.69 × 10^9^/L during induction; leukocytosis during induction occurred in 100 (88.5%) patients with a maximum WBC count of 35.79 × 10^9^/L. Except for three patients developed early death in this group, 75 patients received cytarabine alone, 7 received mitoxantrone alone, 7 received daunorubicin alone, 8 received cytarabine and mitoxantrone, 13 received cytarabine and daunorubicin. Thirty‐three of 110 patients received hydroxycarbamide with a mean cumulative dose of less than 7.0 g before the initiation of anthracyclines/cytarabine. Etoposide was initiated in patients with a WBC count of 4.21–9.82 × 10^9^/L during induction. Details about cytoreduction therapies can be found in Supplementary Results. In order to illustrate the role of cytoreductive therapy in the prognosis of low‐risk APL patients, indicators of treatment response, ED and relapse were analysed among the groups (Tables 1 and 2).

**TABLE 1:** Responses and outcomes of different cytoreduction during induction therapy.

	Cytoreductive therapy (*n* = 282)				
	None (*n* = 52)	Hydroxycarbamide (*n* = 86)	Anthracyclines or cytarabine (*n* = 113)	Etoposide (*n* = 31)	*p*
Age, years, median (range)	43.5 (13.0–66.0)	40.0 (14.0–79.0)	38.0 (18.0–70.0)	48.0 (17.0–67.0)	0.500
Male, *n* (%)	25 (48.1)	42 (48.8)	66 (58.4)	18 (58.1)	0.437
WBC count at diagnosis, × 10^9^/L, median (range)	1.31 (0.01–7.84)	1.56 (0.34–8.40)	1.70 (0.30–9.79)	1.44 (0.34–9.82)	0.325
PLT count at diagnosis, × 10^9^/L, median (range)	28.5 (3–201)	32.5 (2–189)	28.0 (2–179)	29.0 (2–203)	0.107
*PML‐RARA* type, *n* (%)					0.250
Long	34 (65.4)	55 (64.0)	73 (64.6)	19 (61.3)	
Short	8 (15.4)	14 (16.3)	16 (14.2)	10 (32.3)	
Variant	10 (19.2)	17 (19.8)	24 (21.2)	2 (6.5)	
*FLT3‐ITD* gene positive at diagnosis, *n* (%)	5 (9.6)	13 (15.1)	6 (19.4)	15 (13.3)	0.646
Complex chromosomal abnormalities, *n* (%)	9 (17.3)	12 (14.0)	19 (16.8)	5 (16.1)	0.942
Myeloblast at diagnosis, *n* (%)	21 (40.4)	30 (34.9)	42 (37.2)	13 (41.9)	0.873
*WT1* (%), median (range)	54.4 (1.0–145.5)	51.5 (2.3–556.8)	55.0 (1.1–157.5)	62.3 (1.8–214.7)	0.644
*PRAME* (%), median (range)	7.2 (1.2–696.4)	10.1 (0.3–398.0)	7.8 (0.5–450.0)	18.2 (0.9–268.3)	0.890
ED, *n* (%)	1 (1.9)	2 (2.3)	3 (2.6)	0 (0.0)	0.840
Leukocytosis during induction, *n* (%)	2 (3.8)	43 (50.0)	100 (88.5)	20 (64.5)	–
Differentiation syndrome, *n* (%)	0 (0.0)	19 (22.1)	26 (23.0)	4 (12.9)	–

	Cytoreductive therapy (*n* = 276)				
	None (*n* = 51)	Hydroxycarbamide (*n* = 84)	Anthracyclines or cytarabine (*n* = 110)	Etoposide (*n* = 31)	*p*
CR^a^, *n* (%)	51 (100.0)	84 (100.0)	110 (100.0)	31 (100.0)	–
Time to CR, days, median (range)	37 (30–41)	38 (28–37)	36 (29–49)	35 (33–39)	0.393
*PML‐RARA* ≥ 6.5% at end of induction	14 (27.5)	22 (26.2)	17 (15.5)	0 (0.0)	0.003

Abbreviations: CMR, complete molecular remission; CR, complete remission; ED, early death. *PML‐RARA*, promyelocytic leukaemia retinoic acid receptor alpha.

^a^ CR after induction therapy .

**TABLE 2:** Relapse of different cytoreduction during induction therapy.

	Cytoreductive therapy (*n* = 267)				*p* ^c^
	None (*n* = 51)	Hydroxycarbamide (*n* = 79)	Anthracyclines or cytarabine (*n* = 106)	Etoposide (*n* = 31)	
CMR^a^	50 (98.0)	78 (98.7)	106 (100.0)	31 (100.0)	0.508
Relapse (*n*)	3	9	2	0	0.017
2‐year cumulative relapse (%)	3.962	8.861	1.887	–	0.033
2‐year relapse‐free survival (%)	96.038	91.139	98.113	–	0.033
Time to relapse^b^, months, median (range)	24.4 (17.1–36.7)	19.8 (6.2–52.9)	14.3 (10.1, 18.4)	–	0.595
Follow‐up time, months, median (range)	64.0 (17.0–102.0)	68.0 (6.2–101.0)	57.0 (10.0–100.0)	17.0 (9.0–24.0)	0.165

Abbreviations: CMR, complete molecular remission; CR, complete remission.

^a^ CMR after the fourth round of consolidation therapy.

^b^ The time to haematological/molecular/extramedullary relapse after a haematological CR.

^c^ *p* values of CMR and relapse were among the four groups. *p*‐values of relapse, 2‐year cumulative relapse, 2‐year relapse‐free survival, time to relapse and follow‐up time were among three groups of no cytoreduction group, the hydroxycarbamide treatment group and the anthracyclines/cytarabine treatment group.

There were no significant differences in the incidences of CR and CMR after consolidation therapy, or the time to CR after induction therapy among the groups (Tables 1 and 2). No difference was observed in the incidence of ED among the four groups (Table 1). Our previous study found that a *PML‐RARA* transcript level of 6.5% or more at the end of induction therapy was associated with a subsequent risk of relapse.^5^ Compared to patients with no cytoreduction therapy or only hydroxycarbamide during induction therapy, the incidence of *PML‐RARA* transcript levels of ≥6.5% was significantly decreased at the end of induction therapy in those who received anthracyclines/cytarabine and etoposide (27.5% vs. 26.2% vs. 15.5% vs. 0%, *p* = 0.003, Table 1). Notably, the hydroxycarbamide treatment group did not decrease the relapse rate compared to the no cytoreduction group (11.4% vs. 5.9%, *p* = 0.289), neither nor the 5‐year RFS (88.145% vs. 93.752%, *p* = 0.292). Compared with the hydroxycarbamide group, the anthracyclines/cytarabine treatment group showed improved 5‐year RFS (88.145% vs. 98.113%, *p* = 0.008, Figure 2C). Data from the etoposide treatment group was reported in the Supplementary Results because of the short follow‐up time (median 17.0 months, range 9.0–24.0 months).

### 3.4 Risk factors of relapse

In the univariate analysis, expressions of cluster of differentiation (CD) 34, CD56, CD64 and CD2, *PML‐RARA* type, co‐expression of *FLT3‐ITD*, complex karyotype, Wilms tumour 1 (*WT1*), preferentially expressed antigen in melanoma (*PRAME*), and leukocytosis during induction therapy were not associated with relapse (Table 3). Cytoreductive chemotherapies were associated with RFS, the anthracyclines/cytarabine group showed improved RFS versus the hydroxycarbamide group (Figure 2C, 5‐year RFS, 88.145% vs. 98.113%, *p* = 0.008). Morphologically, myeloblasts in bone marrow at diagnosis were associated with RFS (Figure 2D, 5‐year RFS, 90.126% vs. 96.476%, *p* = 0.0201, Supplementary Results). Besides, *PML‐RARA* of 6.5% or more at the end of induction therapy was also associated with RFS (Figure 2E, 5‐year RFS, 83.019% vs. 97.067%, *p* < 0.0001).

**TABLE 3:** Univariate analysis of relapse.

Risk factors	Univariate analysis				
	No relapse (*n* = 253)	Relapse (*n* = 14)	2‐year RFS (%)	HR (95% CI)	*p*
Myeloblast at diagnosis, *n* (%)				3.227 (1.080–9.636)	0.036
Yes	91 (36.0)	9 (64.3)	91.4		
No	162 (64.0)	5 (35.7)	97.4		
CD34, *n* (%)				0.431 (0.056–3.296)	0.417
Yes	41 (16.2)	1 (7.1)	97.4		
No	212 (83.8)	13 (92.9)	96.3		
CD56, *n* (%)				1.248 (0.279–5.583)	0.772
Yes	30 (11.9)	2 (14.3)	93.6		
No	223 (88.1)	12 (85.7)	97.3		
CD64, *n* (%)				0.996 (0.223–4.449)	0.996
Yes	216 (85.4)	12 (85.7)	92.0		
No	37 (14.6)	2 (14.3)	96.4		
CD2, *n* (%)				1.953 (0.435–8.775)	0.382
Yes	25 (9.9)	2 (14.3)	92.1		
No	228 (90.1)	12 (85.7)	97.0		
*PML‐RARA* type, *n* (%)					
Long	168 (66.4)	8 (57.1)	97.0	–	–
Short	38 (15.0)	4 (28.6)	89.1	0.396 (0.119–1.325)	0.133
Variant	47 (18.6)	2 (14.3)	95.8	0.330 (0.060–1.818)	0.203
*FLT3‐ITD* gene positive at diagnosis, *n* (%)				2.180 (0.601–7.833)	0.232
Yes	30 (11.9)	3 (21.4)	90.5		
No	223 (88.1)	11 (78.6)	97.3		
Complex chromosomal abnormalities, *n* (%)				0.480 (0.063–3.669)	0.479
Yes	37 (14.6)	1 (7.1)	97.1		
No	216 (85.4)	13 (92.9)	96.4		
Leukocytosis during induction therapy, *n* (%)				1.683 (0.528–5.368)	0.179
Yes	151 (59.7)	10 (71.4)	95.5		
No	102 (40.3)	4 (28.6)	95.9		
Cytoreductive therapy, *n* (%)					
None	48 (19.0)	3 (21.4)	93.8	–	–
Hydroxycarbamide	70 (27.7)	9 (64.3)	89.9	0.503 (0.136–1.857)	0.302
Anthracyclines or cytarabine	104 (41.1)	2 (14.3)	98.1	0.162 (0.035–0.749)	0.020
*PML‐RARA* ≥ 6.5% at end of induction				7.040 (2.359–21.012)	<0.001
Yes	44 (17.4)	9 (64.3)	83.0		
No	209 (82.6)	5 (35.7)	98.5		

Abbreviations: CD, cluster of differentiation; CI, confidence interval; *FLT3‐ITD*, FMS‐like tyrosine kinase 3‐internal tandem duplication; HR, hazard ratio; *PML‐RARA*, promyelocytic leukaemia retinoic acid receptor alpha; *PRAME*, preferentially expressed antigen in melanoma; RFS, relapse‐free survival; *WT1*, Wilms tumour 1.

**FIGURE 3:**
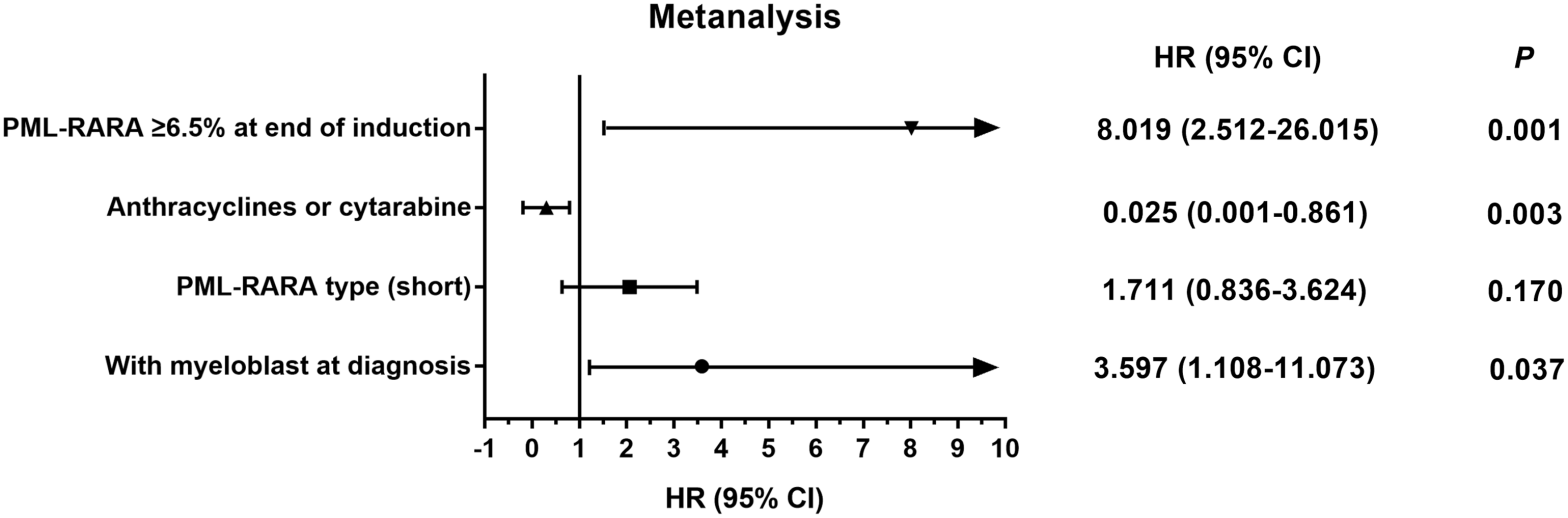
Multivariate analysis of relapse. CI, confidence interval; HR, hazard ratio; *PML‐RARA*, promyelocytic leukaemia retinoic acid receptor alpha.

Further multivariate Cox regression analysis revealed that myeloblasts in bone marrow at diagnosis [hazard ratio (HR) 3.597, 95% confidence interval (CI) 1.108–11.073, *p* = 0.037] and *PML‐RARA* of ≥6.5% (HR 8.019, 95% CI 2.512–26.015, *p* = 0.001) were associated with a subsequent risk of relapse (Figure 3). Anthracyclines/cytarabine cytoreductive treatment (HR 0.025, 95% CI 0.001–0.861, *p* = 0.003) was the only factor that reduced the relapse rate.

### 3.5 Adverse events

The main adverse effects are listed in Table 4. Adverse effects mainly consisted of neutropenia, thrombocytopenia, liver injury and infection. Forty‐two patients (14.9%) had Grade 1–4 diarrhoea. Sixteen (5.6%) patients in the ATRA‐RIF group were switched to ATRA‐ATO due to Grade 3–4 diarrhoea or hemafecia. In total, 183 patients (64.9%) had Grade 1–4 liver injury. Grade 3–4 liver injury was reported in 30 (11.2%) of 267 patients and the toxic effects resolved with temporary discontinuation of arsenic, ATRA or both.

**TABLE 4:** Adverse events reported in treatment groups.

Adverse events	Cytoreductive therapy (*n* = 267)			
	None (*n* = 51)	Hydroxycarbamide (*n* = 79)	Anthracyclines or cytarabine (*n* = 106)	Etoposide (*n* = 31)
Neutropenia (grade 3–4 lasting > 15 days)	16 (31.3)	20 (25.3)	25 (23.5)	6 (19.4)
Thrombocytopenia (grade 3–4 lasting > 15 days)	23 (45.1)	32 (40.5)	42 (39.6)	13 (41.9)
Liver injury (grade 3–4)	5 (9.8)	10 (12.7)	15 (14.2)	0 (0)
Kidney injury (grade 3–4)	1 (2.0)	0 (0)	4 (4.8)	0 (0)
Gastrointestinal toxicity (grade 3–4)	3 (5.9)	5 (6.3)	7 (6.6)	1 (3.2)
Oral mucositis (grade 3–4)	0 (0)	1 (1.3)	2 (1.9)	0 (0)
Infection	18 (35.3)	23 (29.1)	40 (37.7)	10 (32.3)
Prolongation of the QTc interval	4 (7.8)	5 (6.3)	9 (8.4)	2 (6.5)
Cardiac function (grade 3–4)	0 (0)	1 (1.3)	1 (0.9)	0 (0)

*Note*: Data are *n* (%).

## 4 DISCUSSION

This study sheds light on the crucial role of cytoreductive chemotherapy during induction therapy in the prognosis of low‐risk APL in the ATRA plus arsenicals era. APL has become curable under ATRA and arsenicals in low‐risk patients.^17^ However, there are thorny issues, such as ED and relapse, which are yet to be addressed. After administration of ATRA and arsenicals, the WBC count will typically increase and may lead to DS, which is one of the main reasons of ED and occurs in 11%–28% of patients with low‐risk APL.^2^, ^4^, ^7^ Our study revealed 58.5% Leukocytosis during induction, 17.4% DS, and 2.1% ED. Cytoreductive chemotherapy may partially prevent or control the risks of DS and still plays a vital role in induction therapy.

Despite the Sanz score being widely used for identifying the risk of APL, additional genetic mutations as exemplified by *FLT3‐ITD* or *TKD*, and the expression of CD34 and/or CD56 antigens are also considered to have prognostic relevance,^2^, ^4^, ^11^, ^18^, ^19^, ^20^, ^21^, ^22^, ^23^ whereas the explicit clinical and molecular parameters to predict relapse remains ambiguous with the first‐line use of ATRA and arsenicals. In the study, we reported that myeloblasts in bone marrow at diagnosis were associated with a subsequent risk of relapse. However, the exact mechanism of this association remains unclear. We speculated that the case with myeloblasts has a more malignant character because of a more primitive stage of promyelocytes versus myeloblasts. Dayton et al. reported that neoplastic hypergranular or microgranular promyelocytes with indented or bivalve nuclei predominated at diagnosis in all patients. Most patients had undifferentiated blasts at relapse and/or hypergranular blast equivalents with round to oval nuclei.^24^ It suggested that morphologic features of relapsed APL overlap with other types of acute myeloid leukaemia with myeloblasts. The clear mechanism between myeloblasts at diagnosis and relapse requires further study.

In the study, patients who received anthracyclines or cytarabine as cytoreduction had a decreased rate of *PML‐RARA* of 6.5% or more at the end of induction therapy, and improved RFS compared to patients with hydroxycarbamide during induction therapy. To our knowledge, research on ATRA‐arsenicals ± cytoreductive therapy is not available to date. Conclusions about the role of chemotherapy in the induction therapy of APL were drawn from studies about ATRA + chemotherapy. That's why the aim of our study is to explore the potential role of cytoreduction during induction therapy in the ATRA plus arsenicals era. In our cohort, 14 patients (5.2%) relapsed during follow‐up, with a 5‐year cumulative relapse incidence of 5.9%. Myeloblasts in bone marrow at diagnosis, *PML‐RARA* of 6.5% or more and anthracyclines/cytarabine cytoreductive treatment were associated with a subsequent risk of relapse. According to our Cox proportional hazard regression model, cytoreductive chemotherapy, which had the most weight, was shown to reduce the risk of relapse. Chemotherapy‐associated secondary myeloid malignancies and more early and late adverse events need long‐term observation.

This study explored an all‐oral regimen of etoposide as cytoreductive therapy combined with RIF and ATRA for low‐risk APL. NCCN guidelines recommend anthracycline‐based chemotherapy as one part of induction therapy for newly diagnosed APL to reduce WBC count. However, the guidelines do not elaborate on when to initiate chemotherapy. Similarly, there is a lack of studies on the optimal timing of chemotherapy during induction treatment.^25^ de Botton et al suggested that early addition of chemotherapy with low WBC counts significantly reduced the incidence of ATRA syndrome during induction therapy.^26^ Here we recommended that oral etoposide, with a relatively slow effect on cytoreduction, was initiated to reduce tumour load as early as possible in order to reduce the rate of ED and ensure the safety of induction therapy. The role of etoposide as cytoreduction therapy in lowering relapse and improving prognosis needs to be verified by larger sample studies and a longer follow‐up period. We are conducting a randomized, controlled clinical trial comparing oral etoposide with intravenous infusion of daunorubicin as cytoreduction plus RIF‐ATRA for induction therapy in low‐risk APL patients (NCT05832320).

In summary, the present study indicated that cytoreductive chemotherapy had a dual effect of both cytoreduction and prevention of relapse in low‐risk APL. Further studies should be explored to confirm the role and its mechanisms of chemotherapies in low‐risk APL, especially for those with WBC counts more than 4 × 10^9^/L during induction therapy. Oral etoposide as cytoreduction therapy combined with oral ATRA plus RIF is expected to be a regimen with low relapse, good tolerance and great convenience. A prospective, randomized, controlled, interventional study on oral etoposide versus intravenous daunorubicin for cytoreductive chemotherapy in induction therapy in patients with low‐risk acute promyelocytic leukaemia is now going on.

## AUTHOR CONTRIBUTIONS

**Xiaolu Zhu:** Data curation (equal); formal analysis (lead); investigation (equal); methodology (lead); writing – original draft (lead). **Feifei Tang:** Data curation (equal); investigation (equal). **Wenjing Yu:** Data curation (equal); investigation (equal). **Xiaosu Zhao:** Data curation (equal); investigation (equal). **Yazhen Qin:** Data curation (equal); investigation (equal). **Qian Jiang:** Data curation (equal); investigation (equal). **Xiaojun Huang:** Supervision (lead); validation (equal); writing – review and editing (equal). **Hao Jiang:** Conceptualization (lead); project administration (lead); validation (equal); writing – review and editing (equal).

## FUNDING INFORMATION

This work was supported by the Peking University People's Hospital Research and Development Foundation (No. RDL2022‐05 and RDH2021‐11), and the National Key Research and Development Program of China (No. 2021YFC2500300).

## CONFLICT OF INTEREST STATEMENT

The authors declare to have no competing interests.

## PATIENT CONSENT STATEMENT

A written informed consent was obtained from all the patients on admission, with which the bone marrow, blood and other samples might be used for scientific research but did not relate to patient's privacy.

## Supporting information


**Data S1:** Supporting Information.

## Data Availability

The data that support the findings of this study are available from the corresponding author upon reasonable request.

## References

[1] Platzbecker U , Avvisati G , Cicconi L , et al. Improved outcomes with retinoic acid and arsenic trioxide compared with retinoic acid and chemotherapy in non‐high‐risk acute promyelocytic leukemia: final results of the randomized Italian‐German APL0406 trial. J Clin Oncol. 2017;35(6):605‐612. 27400939 10.1200/JCO.2016.67.1982

[2] Lo‐Coco F , Avvisati G , Vignetti M , et al. Retinoic acid and arsenic trioxide for acute promyelocytic leukemia. N Engl J Med. 2013;369(2):111‐121. 23841729 10.1056/NEJMoa1300874

[3] Burnett AK , Russell NH , Hills RK , et al. Arsenic trioxide and all‐trans retinoic acid treatment for acute promyelocytic leukaemia in all risk groups (AML17): results of a randomised, controlled, phase 3 trial. Lancet Oncol. 2015;16(13):1295‐1305. 26384238 10.1016/S1470-2045(15)00193-X

[4] Zhu HH , Wu DP , Du X , et al. Oral arsenic plus retinoic acid versus intravenous arsenic plus retinoic acid for non‐high‐risk acute promyelocytic leukaemia: a non‐inferiority, randomised phase 3 trial. Lancet Oncol. 2018;19(7):871‐879. 29884593 10.1016/S1470-2045(18)30295-X

[5] Tang FF , Lu SY , Zhao XS , et al. PML‐RARA transcript levels at the end of induction therapy are associated with prognosis in non‐high‐risk acute promyelocytic leukaemia with all‐trans retinoic acid plus arsenic in front‐line therapy: long‐term follow‐up of a single‐centre cohort study. Br J Haematol. 2021;195(5):722‐730. 34405393 10.1111/bjh.17752

[6] McClellan J , Kohrt H , Coutre S , et al. Treatment advances have not improved the early death rate in acute promyelocytic leukemia. Haematologica. 2012;97(1):133‐136. 21993679 10.3324/haematol.2011.046490 PMC3248942

[7] Abaza Y , Kantarjian H , Garcia‐Manero G , et al. Long‐term outcome of acute promyelocytic leukemia treated with all‐trans‐retinoic acid, arsenic trioxide, and gemtuzumab. Blood. 2017;129(10):1275‐1283. 28003274 10.1182/blood-2016-09-736686 PMC5413297

[8] O'Donnell MR . Risk stratification and emerging treatment strategies in acute myeloid leukemia. J Natl Compr Cancer Netw. 2013;11(5 Suppl):667‐669. 10.6004/jnccn.2013.0197 23704239

[9] Zhu HH , Hu J , Lo‐Coco F , Jin J . The simpler, the better: oral arsenic for acute promyelocytic leukemia. Blood. 2019;134(7):597‐605. 31113776 10.1182/blood.2019000760

[10] National Comprehensive Cancer Network (NCCN) . NCCN clinical practice guidelines in oncology acute myeloid leukemia. Version 1.2021. http://www.nccn.org

[11] Iaccarino L , Ottone T , Alfonso V , et al. Mutational landscape of patients with acute promyelocytic leukemia at diagnosis and relapse. Am J Hematol. 2019;94(10):1091‐1097. 31292998 10.1002/ajh.25573

[12] Ades L , Guerci A , Raffoux E , et al. Very long‐term outcome of acute promyelocytic leukemia after treatment with all‐trans retinoic acid and chemotherapy: the European APL Group experience. Blood. 2010;115(9):1690‐1696. 20018913 10.1182/blood-2009-07-233387

[13] Lu SY , Wen‐Jing L , Lou R , et al. Oral etoposide combined with oral arsenic plus retinoic acid for two cases with newly diagnosed high‐risk acute promyelocytic leukemia during COVID19 pandemic. Leuk Res Rep. 2021;16:100258. 34367907 10.1016/j.lrr.2021.100258 PMC8326808

[14] Chinese Society Of Hematology CMAC . [Chinese guidelines for diagnosis and treatment of acute promyelocytic leukemia (2018)] [article in Chinese]. Zhong Hua Xue Ye Xue Za Zhi. 2018;39(3):179‐183. 10.3760/cma.j.issn.0253-2727.2018.03.002 PMC7342995 29562460

[15] Montesinos P , Bergua JM , Vellenga E , et al. Differentiation syndrome in patients with acute promyelocytic leukemia treated with all‐trans retinoic acid and anthracycline chemotherapy: characteristics, outcome, and prognostic factors. Blood. 2009;113(4):775‐783. 18945964 10.1182/blood-2008-07-168617

[16] Sanz MA , Montesinos P , Rayon C , et al. Risk‐adapted treatment of acute promyelocytic leukemia based on all‐trans retinoic acid and anthracycline with addition of cytarabine in consolidation therapy for high‐risk patients: further improvements in treatment outcome. Blood. 2010;115(25):5137‐5146. 20393132 10.1182/blood-2010-01-266007

[17] Ma YF , Lu Y , Wu Q , et al. Oral arsenic and retinoic acid for high‐risk acute promyelocytic leukemia. J Hematol Oncol. 2022;15(1):148. 36258250 10.1186/s13045-022-01368-3 PMC9578225

[18] Montesinos P , Rayon C , Vellenga E , et al. Clinical significance of CD56 expression in patients with acute promyelocytic leukemia treated with all‐trans retinoic acid and anthracycline‐based regimens. Blood. 2011;117(6):1799‐1805. 21148082 10.1182/blood-2010-04-277434

[19] Yoshii M , Ishida M , Yoshida T , et al. Clinicopathological features of acute promyelocytic leukemia: an experience in one institute emphasizing the morphological and immunophenotypic changes at the time of relapse. Int J Clin Exp Pathol. 2013;6(10):2192‐2198. 24133598 PMC3796242

[20] Teng‐Fei S , Diyaer A , Hong‐Ming Z , et al. Evolving of treatment paradigms and challenges in acute promyelocytic leukaemia: a real‐world analysis of 1105 patients over the last three decades. Transl Oncol. 2022;25:101522. 36075113 10.1016/j.tranon.2022.101522 PMC9465437

[21] Fasan A , Haferlach C , Perglerova K , et al. Molecular landscape of acute promyelocytic leukemia at diagnosis and relapse. Haematologica. 2017;102(6):e222‐e224. 28341736 10.3324/haematol.2016.162206 PMC5451348

[22] Maenhout TM , Moreau E , Van Haute I , et al. Minimal Coexpression of CD34+/CD56+ in acute promyelocytic leukemia is associated with relapse. Am J Clin Pathol. 2015;144(2):347‐351. 26185322 10.1309/AJCPBS3W1RJDGPZU

[23] Chendamarai E , Balasubramanian P , George B , et al. Role of minimal residual disease monitoring in acute promyelocytic leukemia treated with arsenic trioxide in frontline therapy. Blood. 2012;119(15):3413‐3419. 22374701 10.1182/blood-2011-11-393264

[24] Dayton VJ , McKenna RW , Yohe SL , et al. Relapsed acute promyelocytic leukemia lacks “classic” leukemic promyelocyte morphology and can create diagnostic challenges. Am J Clin Pathol. 2017;147(1):69‐76. 28108472 10.1093/ajcp/aqw202

[25] Xu F , Yin CX , Wang CL , et al. Influence of initiation time and white blood cell count on the efficacy of cytotoxic agents in acute promyelocytic leukemia during induction treatment. Biomed Rep. 2018;9(3):227‐232. 30271598 10.3892/br.2018.1120 PMC6158397

[26] de Botton S , Chevret S , Coiteux V , et al. Early onset of chemotherapy can reduce the incidence of ATRA syndrome in newly diagnosed acute promyelocytic leukemia (APL) with low white blood cell counts: results from APL 93 trial. Leukemia. 2003;17(2):339‐342. 12592333 10.1038/sj.leu.2402807

