# Supplementary material for: Cytoreductive chemotherapy in induction therapy plays a key role in the prognosis of patients with low‐risk acute promyelocytic leukaemia
Source: J Cell Mol Med. 2024 May 20;28(10):e18252. doi: 10.1111/jcmm.18252 (PMC11103457; doi:10.1111/jcmm.18252)
Supplement: Supplementary file 1 — Data S1: Supporting Information. [file JCMM-28-e18252-s001.docx]

**Supplementary** **materials**

**Results**

**Cytoreduction therapy during the induction therapy**

For the hydroxycarbamide treatment group, the cumulative mean dose of hydroxycarbamide was 19.5 g (range, 12.5-28.5 g) during the induction therapy. For the anthracyclines/cytarabine treatment group, the subgroups here included: cytarabine alone, mitoxantrone alone, daunorubicin alone, cytarabine plus mitoxantrone, and cytarabine plus daunorubicin groups. In the cytarabine alone group, the cumulative mean dose was 1600 mg (range, 800-3300 mg) in during the induction therapy. In the mitoxantrone alone group, the mean cumulative dose was 16 mg (range, 8-32 mg) during the induction therapy. In the daunorubicin alone group, the mean cumulative dose was 100 mg (range, 80-160 mg) during the induction therapy. In the cytarabine plus mitoxantrone combined group, the mean cumulative dose of cytarabine was 1000 mg (range, 400-1800 mg), the mean cumulative dose of mitoxantrone was 10 mg (range, 4-20 mg). In the cytarabine plus daunorubicin combined group, the mean cumulative dose of cytarabine was 1200 mg (range, 400-2000 mg), the mean cumulative dose of daunorubicin was 80 mg (range, 40-120 mg). Two patients in the anthracyclines/cytarabine treatment group relapsed throughout follow-up with the cytoreductive therapy of cytarabine alone. No relapse was found in the anthracyclines treatment group.

**The etoposide cytoreduction treatment**

In the study, 31 patients were administered with etoposide (11.0%). Etoposide was initiated to patients with a WBC count of 4.21-9.82×10^9^/L on days 6-13 of induction therapy. Leukocytosis during induction was occurred in 20 (64.5%) patients with the maximum WBC count of 21.95×10^9^/L. The cumulative mean dose of etoposide was 706.5 mg (range, 150-1700 mg) during the induction therapy. The median follow-up of this group is 17.0 months, range 9.0-24.0 months. All of patients with cytoreduction therapy of etoposide have complete molecular remission (CMR) after the fourth rounds of consolidation therapy. No early death (ED) and no relapse happened during the follow-up. In terms of adverse events, five of 31 patients (16.1%) in the etoposide treatment group had Grade 1-2 hepatoxic effects, one (3.2%) had Grade 1 nephrotoxic effects. No toxic effects of nervous system, circulatory system and respiratory system were observed in the group.

**Risk factors of relapse**

Myeloblasts were defined morphologically. The shape of myeloblast is round or oval, with very little cell mass, uniform sky blue color, and no granules. The nuclei are round and mostly offset, the nuclear chromatin is fine, the granules are distributed like gossamer, the nuclear perimeter is obvious, and the nucleolus is Sizes vary. The bone marrow morphology of APL is dominated by abnormal promyelocytes with increased granules, most of which are not accompanied by myeloblasts. Morphologically, myeloblasts in bone marrow at diagnosis was associated with RFS in our study.

**Supplementary Table 1. Baseline characteristics of low-risk APL patients.**

| Characteristic | Value |
| --- | --- |
| Age, years, median (range) | 40.0 (13.0-79.0) |
| Gender, n (%) |  |
| Male | 151 (53.5) |
| Female | 131 (46.5) |
| WBC count, × 10⁹/L, median (range) | 1.54 (0.01-9.82) |
| PLT count, × 10⁹/L, median (range) | 30 (2-203) |
| *PML-RARA* type, n (%) |  |
| Long | 181 (64.2) |
| Short | 48 (17.0) |
| Variant | 53 (18.8) |
| *FLT3-ITD* gene positive at diagnosis, n (%) |  |
| Yes | 39 (13.8) |
| No | 243 (86.2) |
| Complex chromosomal abnormalities,n (%) |  |
| Yes | 45 (16.0) |
| No | 237 (84.0) |
| Myeloblast in bone marrow (%), median (range) | 2.1 (0-48.5) |
| *WT1*(%), median (range) | 55.9 (1.0-556.8) |
| *PRAME*(%), median (range) | 11.1 (0.3-696.4) |

WBC, white blood cell; PLT, Platelets; *FLT3-ITD*, FMS-like tyrosine kinase 3-internal tandem duplication; *PML-RARA*, promyelocytic leukemia retinoic acid receptor alpha; *WT1*, Wilms tumor 1; *PRAME*, preferentially expressed antigen in melanoma; APL, acute promyelocytic leukemia.

Figure S1. Alcian blue staining of MSCs following chondrogenic differentiation with the treatment of CA For 0, 7, and 14 days. Scale bar, 1 mm.
